# Cryopreservative solution using rakkyo fructan as cryoprotectant

**DOI:** 10.1186/1753-6561-7-S6-P105

**Published:** 2013-12-04

**Authors:** Satoshi Terada, Shinya Mizui, Yasuhito Chida, Masafumi Shimizu, Akiko Ogawa, Takeshi Ohura, Kyo-ichi Kobayashi, Saori Yasukawa, Nobuyuki Moriyama

**Affiliations:** 1 Department of Applied Chemistry and Biotechnology, University of Fukui, 3-9-1 Bunkyo, Fukui, 910-8507, Japan; 2 Department of Chemistry and Biochemistry, Suzuka National College of Technology, Shiroko-cho, Suzuka, 510-0294, Japan; 3 Fukui Prefectural Food Process, 1-1-1 Maruoka-cho-tubonouchi, Sakai, 910-0343, Japan; 4 ELLE ROSE CO., Ltd., 4-200 Saburoumaru, Fukui, 910-0033, Japan

## Introduction

Cryopreservation of the cells allows great flexible application for cell therapy, as well as industrial production of biologics such as antibody therapeutics. Conventionally, cryopreservative solution contains both of fetal bovine serum (FBS) and dimethyl sulfoxide (DMSO) as a cryoprotectant [1]. However, both of them have problems. FBS frequently induces differentiation of stem cells and so it should not be used for cell therapy. Additionally, FBS has serious concern about zoonotic infections such as abnormal prions, pathogen of bovine spongiform encephalopathy (BSE) [2,3], indicating necessity of FBS-free cryopreservative solution. DMSO has cytotoxicity and often induces stem cells to differentiate [3]. Therefore, it is necessary to reduce the concentration of DMSO in cryoprotectant solution. In this study, we report that rakkyo fructan, plant-derived polysaccharide, significantly improved the viability of the cells frozen in DMSO-free solution.

## Materials and methods

### Cell line and culture condition

A mouse hybridoma 2E3-O [4] was used for this study. 2E3-O was cultured in ASF104 (Ajinomoto, Tokyo, Japan) with 1 g/L bovine serum albumin (BSA, Wako pure chemical industries, Osaka, Japan).

### Polysaccharides and cryopreservative solution

Rakkyo fructan was purified by the method in previous study [5]. Low molecular weight inulin and high one were produced by Fuji Nihon Seito Co. (Tokyo, Japan). Levan was purchased from Wako pure chemical industries. Each polysaccharide was solved in phosphate buffer saline (PBS). FBS containing 10% DMSO was used as positive control.

### Cryopreservative procedure

2E3-O cells were pre-cultured until 60-70% confluent before cryopreservation. They were collected by centrifugation, removed the culture supernatant and then suspended in the cryopreservative solution. They were transferred to freezing tubes, placed in a BIOCELL container (Nihon freezer, Tokyo, Japan), frozen and stored at -80°C for several days.

### Thawing procedure and re-culture

Stored cells were defrosted at 37°C rapidly then transferred to the culture medium. The defrosted cells were centrifuged in order to the cryopreservative solution. Collected cells were suspended by the culture medium again. A part of them was stained with trypan blue exclusion method and counted with hemocytometer. The other one was re-cultured in a multi well plate for several days. After that, grown cells were stained with trypan blue exclusion method and counted with hemocytometer.

## Results and discussion

**Figure 1:**
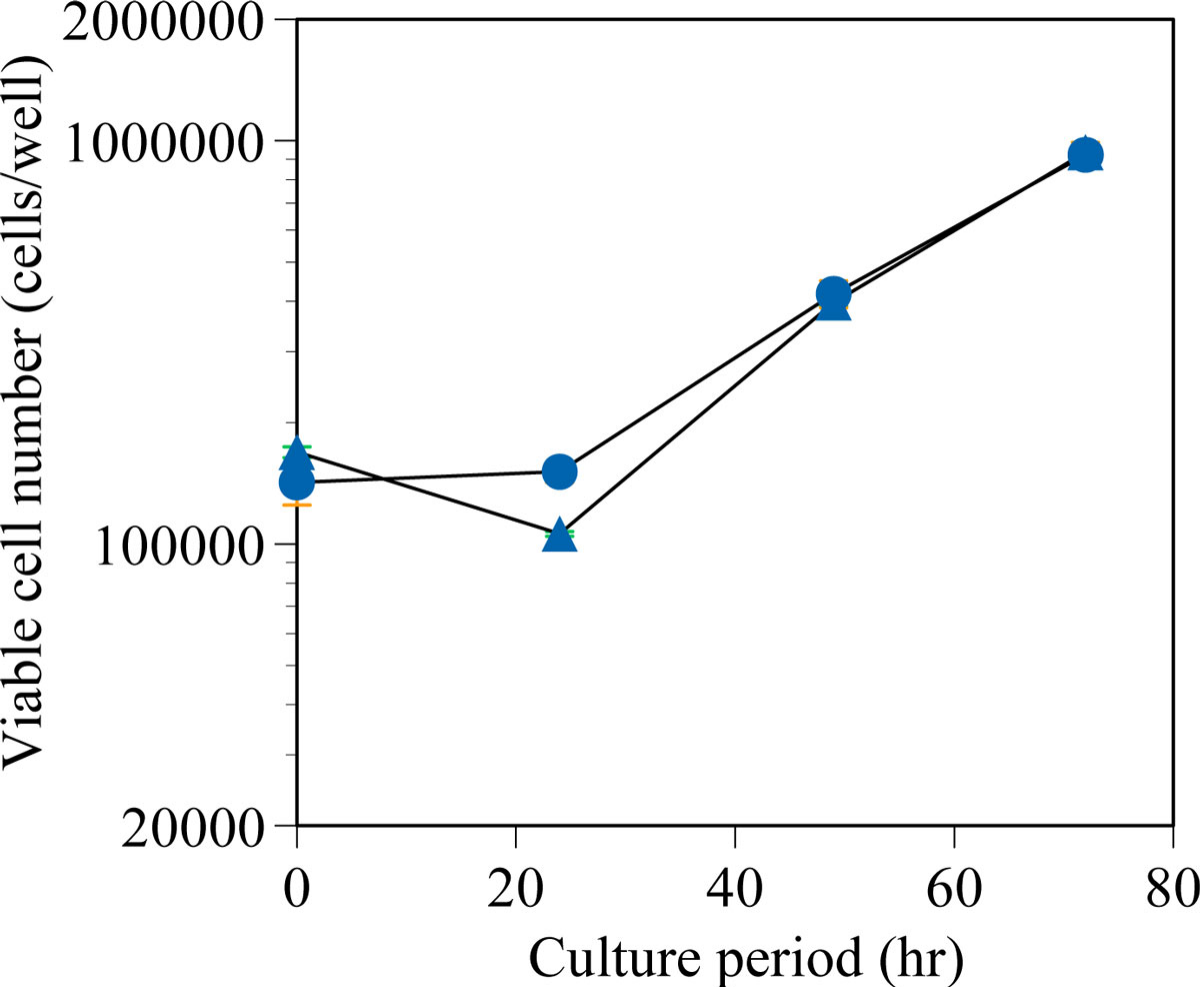
**The time curse of viable cell number after thawing of frozen cells**. 2E3-O cells were stored for three days in 10 w/v% rakkyo fructan (triangles) or 30 w/v% rakkyo fructan (circles). The experiment was four trials.

2E3-O cells stored in 3 w/v%, 10 w/v% or 30 w/v% rakkyo fructan solution. After frozen and thawed in 10 w/v% or 30 w/v% rakkyo fructan solution, 2E3-O cells successfully survived and proliferated (Figure 1). On the other hand, all 2E3-O cells stored in 3 w/v% rakkyo fructan solution were dead (data not shown). This result shows that using rakkyo fructan will be effective for serum-free cryopreservation without DMSO.

To compare the effect of rakkyo fructan on cellular protection, other fructans such as inulin and levan were also used for cryopreservation. Four fructans were different in molecular weight and solubility. Rakkyo fructan and low molecular weight inulin solved in water very much but high molecular weight inulin solved in water up to 10 w/v% and levan dissolved in water. Rakkyo fructan was the highest viable cell number among fructans (Table 1). This result indicates that rakkyo fructan can protect animal cells more effectively than other fructans. Using rakkyo fructan has some advantages: 1) using rakkyo fructan can avoid pathogenic contamination, 2) using rakkyo fructan will not be occurred osmotic change of stored cells because molecular weight of rakkyo fructan is over 10,000 (i.e. 30 w/v% rakkyo fructan is about 0.03 M), and 3) rakkyo fructan is high water soluble, which is easy to use.

**Table 1:** Viable cell number of 2E3-O cells after frozen-thawing process.

Cryopreservative solution	Mean degree of polymerization	Viable cell number (×10^6^)
30 w/v% rakkyo fructan	390	99.5
30 w/v% inulin (low molecular weight)	16	64.5
10 w/v% inulin (high molecular weight)	19	5.0
1 w/v% levan	1000	0.2
Positive control	-	111

2E3-O cells were stored for three days. 1.18 × 10^6 ^cells were frozen.

## Conclusion

In conclusion, the freezing media using rakkyo fructan will be extensively used to protect animal cells against freezing stress without DMSO.
